# Novel mutations in the *WFS1* gene are associated with Wolfram syndrome and systemic inflammation

**DOI:** 10.1093/hmg/ddab040

**Published:** 2021-03-09

**Authors:** Eleonora Panfili, Giada Mondanelli, Ciriana Orabona, Maria L Belladonna, Marco Gargaro, Francesca Fallarino, Elena Orecchini, Paolo Prontera, Elisa Proietti, Giulio Frontino, Eva Tirelli, Alberta Iacono, Carmine Vacca, Paolo Puccetti, Ursula Grohmann, Susanna Esposito, Maria T Pallotta

**Affiliations:** Department of Medicine and Surgery, University of Perugia, Perugia, 06132, Italy; Department of Medicine and Surgery, University of Perugia, Perugia, 06132, Italy; Department of Medicine and Surgery, University of Perugia, Perugia, 06132, Italy; Department of Medicine and Surgery, University of Perugia, Perugia, 06132, Italy; Department of Medicine and Surgery, University of Perugia, Perugia, 06132, Italy; Department of Medicine and Surgery, University of Perugia, Perugia, 06132, Italy; Department of Medicine and Surgery, University of Perugia, Perugia, 06132, Italy; Medical Genetics Unit, University-Hospital “Santa Maria della Misericordia”, Perugia, 06132, Italy; Department of Medicine and Surgery, University of Perugia, Perugia, 06132, Italy; Department of Pediatrics, Diabetes Research Institute, IRCCS San Raffaele Hospital, Milan, 20132, Italy; Department of Pediatrics, Diabetes Research Institute, IRCCS San Raffaele Hospital, Milan, 20132, Italy; Department of Medicine and Surgery, University of Perugia, Perugia, 06132, Italy; Department of Medicine and Surgery, University of Perugia, Perugia, 06132, Italy; Department of Medicine and Surgery, University of Perugia, Perugia, 06132, Italy; Department of Medicine and Surgery, University of Perugia, Perugia, 06132, Italy; Visiting Professor, Department of Pathology, Albert Einstein College of Medicine, Bronx, NY 10461, USA; Pediatric Clinic Pietro Barilla Children’s Hospital, Department of Medicine and Surgery, Università di Parma, Parma, 43126, Italy; Department of Medicine and Surgery, University of Perugia, Perugia, 06132, Italy

## Abstract

Mutations in the *WFS1* gene, encoding wolframin (WFS1), cause endoplasmic reticulum (ER) stress and are associated with a rare autosomal-recessive disorder known as Wolfram syndrome (WS). WS is clinically characterized by childhood-onset diabetes mellitus, optic atrophy, deafness, diabetes insipidus and neurological signs. We identified two novel *WFS1* mutations in a patient with WS, namely, c.316-1G > A (in intron 3) and c.757A > T (in exon 7). Both mutations, located in the N-terminal region of the protein, were predicted to generate a truncated and inactive form of WFS1. We found that although the WFS1 protein was not expressed in peripheral blood mononuclear cells (PBMCs) of the proband, no constitutive ER stress activation could be detected in those cells. In contrast, WS proband’s PBMCs produced very high levels of proinflammatory cytokines (i.e. TNF-α, IL-1β, and IL-6) in the absence of any stimulus. *WFS1* silencing in PBMCs from control subjects by means of small RNA interference also induced a pronounced proinflammatory cytokine profile. The same cytokines were also significantly higher in sera from the WS patient as compared to matched healthy controls. Moreover, the chronic inflammatory state was associated with a dominance of proinflammatory T helper 17 (Th17)-type cells over regulatory T (Treg) lymphocytes in the WS PBMCs. The identification of a state of systemic chronic inflammation associated with WFS1 deficiency may pave the way to innovative and personalized therapeutic interventions in WS.

## Introduction

Wolfram syndrome (WS; OMIM 222300), also known as DIDMOAD syndrome—for involving diabetes insipidus (DI), diabetes mellitus (DM), optic atrophy (OA) and deafness (D)—is an autosomal-recessive disorder usually diagnosed in childhood when non-autoimmune, insulin-dependent DM is associated with OA. Other symptoms include bladder, bowel and temperature regulation dysfunctions, as well as endocrinological, psychiatric and neurological abnormalities (1–4). Death occurs prematurely, with a median age of 30 years, often due to respiratory failure caused by brain stem atrophy.

In 1938, *WFS1* was recognized as the causative gene of this disease. It is mapped on chromosome 4p16 and consists of eight exons, encompassing 33.4 kb of genomic DNA. Exon 1 is non-coding, exons 2–7 are small coding exons, and the largest exon, exon 8, is 2.6 kb long. *WFS1* encodes the 890 amino acid hydrophobic glycoprotein wolframin (WFS1), composed of nine transmembrane segments and localized primarily in the membrane of endoplasmic reticulum (ER) (5). In WS individuals, over 200 distinct *WFS1* mutations have been identified so far, most of which are located in exon 8, i.e. in the region that encodes the transmembrane and C-terminal domain of the protein. Gene variants are heterogeneous and include a multiplicity of missense, non-sense, and frameshift insertions or deletion mutations (5,6). Although the molecular mechanisms are not completely elucidated yet, several lines of evidence suggest that the WFS1 protein is important for the maintenance of ER homeostasis. In fact, in the presence of *WFS1* variants and as observed in experimental knock out model systems, uncontrolled calcium ion release from ER does occur and activation of several factors belonging in the unfolded protein response (UPR) signaling pathway can be observed. These events extensively damage affected cells, mostly pancreatic β-cells and neurons, and lead to cell death (7,8).

In the present study, we identified two novel mutations—i.e. outside exon 8 where the majority of mutations have been described—in the *WFS1* gene of an 11-year old WS patient with DM and initial signs of OA. In peripheral blood mononuclear cells (PBMCs), these mutations translated into a lack of expression of the WFS1 protein but not in constitutive ER stress activation. In contrast, constitutive production (i.e. in the absence of any stimulus) of high levels of proinflammatory cytokines and a high ratio of proinflammatory T helper type 17 (Th17) cells to regulatory T (Treg) cells were detected in the PBMCs of the proband.

## Results

### Identification of *WFS1* novel mutations

**Figure 1:**
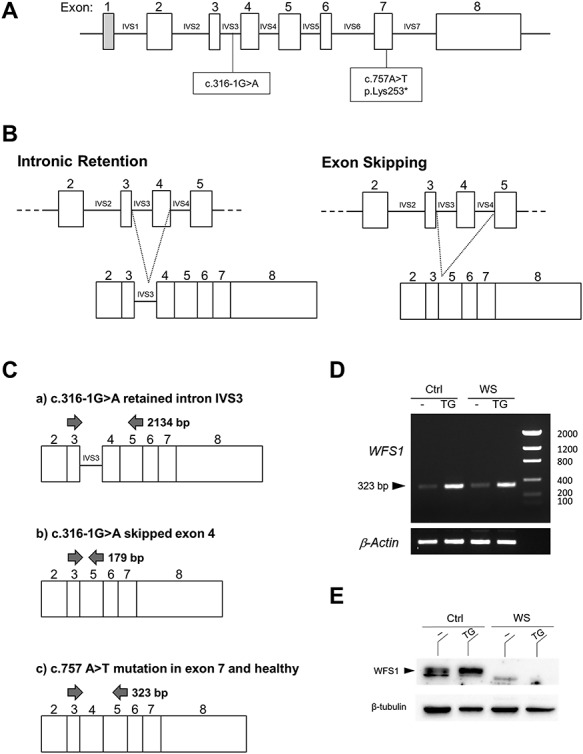
Identification of two novel mutations in the *WFS1* gene and expression of its variants. (**A**) Variants c.316-1G > A and c.757A > T of the *WFS1* gene are located in intron 3 (IVS3) and in exon 7, respectively. The latter is predicted to introduce a stop signal at codon 253 (p.Lys253^*^). (**B**) The c.316-1G > A variant is predicted to cause intron 3 (IV3) retention in the mature transcript, with a reading code slippage (left panel), or exon 4 skipping (right panel). (**C**) PCR strategy to reveal the expression of *WFS1* gene variants altering transcript length in PBMCs. A primer pair annealing on exon 3 and exon 5 was designed such that the IVS3 intronic retention (2134 bp), exon 4 skipping (179 bp), or exon 7 mutation/wild type (323 bp) could be recognized depending on the obtained PCR product. (**D**) End-point PCR with primers annealing on exon 3 and exon 5 and (E) western blot analysis of the WFS1 protein in PBMCs from a healthy donor (Ctrl) and WS patient (WS) cultured for 48 h with or without TG 0.5 μM. β*-*actin and β-tubulin were used as housekeeping gene and loading control, respectively.

A *WFS1* sequencing conducted in the affected WS proband revealed the presence of two heterozygous mutations not previously described, namely, c.316-1G > A and c.757A > T (Fig. 1A). The c.316-1G > A, paternally inherited, was located in intron 3 (IVS3) and caused the loss of the canonic splicing site at the 3′ intronic. This condition may favor the retention of intron 3 and therefore a slippage of the reading code or, alternatively, the skipping of exon 4 in the mature mRNA sequence (Fig. 1B). Intronic retention could be responsible for a stop codon formation leading to a truncated WFS1 protein, whereas exon skipping could produce mRNA coding for an aberrant protein. Although the first condition obtained major score prediction by using the Human Splicing Finder and the Mutation Taster tools, it is plausible that both of these splicing processes may occur. The *WFS1* of the proband also contained the maternally inherited non-sense mutation c.757A > T. Located in exon 7, this mutation is predicted to introduce a stop signal at codon 253 (p.Lys253^*^) and thus would prematurely interrupt a complete translation of the protein.

In order to investigate the possible gene variants expressed in the proband’s PBMCs, we adopted a polymerase chain reaction (PCR) strategy capable of revealing the transcript length encompassing exon 4 by primers designed on exons 3 and 5 (Fig. 1C). Therefore, PBMCs purified from both the proband and healthy donors as a control were cultured for 48 h in the presence or absence of thapsigargin (TG), a *WFS1* and ER stress inducer, and used for transcript analysis. PCR products were sized by comparison with a DNA ladder in a gel electrophoresis, which revealed the presence of a unique 323-bp product in both healthy donors and the WS patient (Fig. 1D), thus demonstrating that, in the proband’s PBMCs, the expressed allele neither contains the IVS3 (c.316-1G > A), nor has exon 4 skipped. In fact, neither a longer (2134 bp, containing retained intron 3), nor a shorter (179 bp, lacking skipped exon 4) product was detected, but rather a 323-bp product consistent with both wild type and exon 7-mutated gene. The absence of detectable transcript from the allele with c.316-1G > A variant argues for a possible mechanism of non-sense-mediated mRNA decay (NMD). The feasibility of amplifying the long intron retention PCR product was validated using PBMC genomic DNA (gDNA) as a template (Supplementary Material, Fig. S1). The signal observed in TG-treated samples, from either control or the WS patient, was increased in accordance with the documented inducer effect of TG on *WFS1* and further demonstrated a high specificity in transcript amplification (Fig. 1D). This induction could be confirmed at the protein level by western blot analysis in healthy controls, whereas, in the proband’s cells, neither the full-length (Fig. 1E) nor the putative p.Lys253^*^-truncated protein (data not shown) were detectable, although western blotting tested even at low molecular weights and exon 7 non-sense mutation preserves the epitope recognized by the detection antibody.

These observations likely suggested that the allele expressed by the WS patient contains the exon 7 non-sense mutation, which, located in the N-terminal of the protein (Supplementary Material, Fig. S2), produces a non-detectable truncated form of WFS1, possibly prone to rapid degradation.

### ER stress markers are not altered in WS proband’s PBMCs

**Figure 2:**
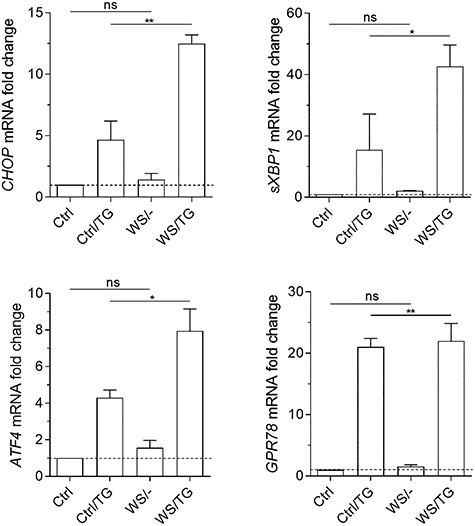
ER stress markers transcripts analyses. Real-time PCR analysis of *CHOP, sXBP1, ATF4, GRP78* transcripts in healthy controls (Ctrl) or PBMCs from the WS patient, untreated or treated with TG at 0.5 μM for 48 h. Data were normalized to the expression of *ACTB* (encoding β-actin) and presented relative to results in healthy controls untreated cells (dotted line, 1-fold). Data were analyzed by one-way ANOVA followed by post hoc Bonferroni’s test. ns, not significant; ^*^P < 0.05; ^*^^*^P < 0.01.

WFS1 is important for maintaining ER homeostasis and its loss of function is responsible for chronic and high ER stress (9,10). In particular, mutation/knock out of WFS1 is associated with ER activation of components of UPR, such as GRP78, CHOP, sXBP1 and ATF4 (7,8). In order to investigate the functional effect of the c.757A > T mutation in *WFS1* in terms of ER stress, we purified PBMCs from the WS proband and healthy subjects as controls. After 48 h of culture, we used real-time PCR to evaluate the transcripts of the main UPR markers, i.e. CHOP, sXBP1, ATF4 and GRP78, in PBMCs either unstimulated or stimulated with TG as ER stress inducer. Intriguingly, in the absence of TG treatment, no significant difference in the expression of ER stress relevant genes could be observed in PBMCs of the WS patient as compared to healthy donors. However, the proband’s cells were more responsive to ER stress induction by TG than the cells of matched control subjects (Fig. 2).

These results indicated that, in the WS patient bearing the c.757A > T mutation, although PBMCs are more prone to ER stress activation, no increased expression of genes coding for CHOP, sXBP1, ATF4, GRP78 could be observed under basal conditions as compared to controls. Therefore, at least in PBMCs, aberrant ER stress may not represent the major pathogenetic mechanism in all WS patients.

### *WFS1* mutation is associated with a high production of proinflammatory cytokines by PBMCs

**Figure 3:**
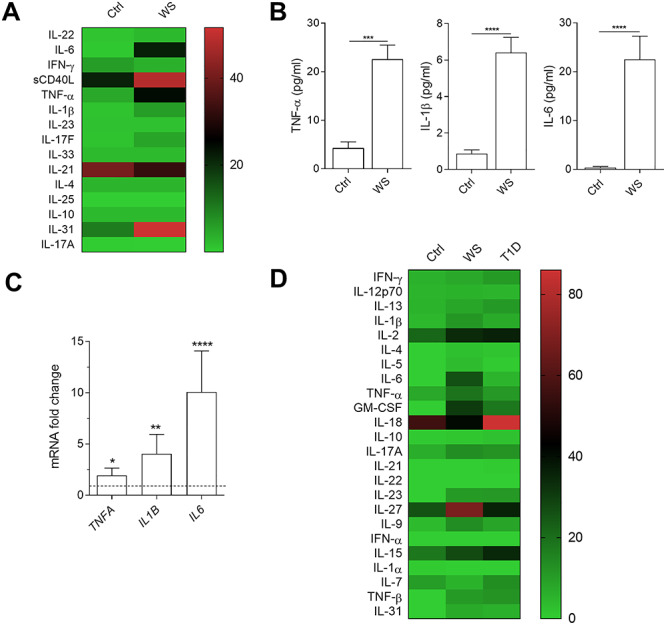
Proinflammatory cytokines production by WS PBMCs. (**A**) Schematic heat map representation of Bio-Plex Pro Human Th17 cytokine panel. The cytokine profile was analyzed in supernatants from PBMCs from healthy donors (Ctrl) and from proband’s PBMCs (WS). The contents of selected cytokines were also analyzed by single-cytokine ELISA test and data were reported in (**B**). (**C**) Real-time PCR analysis of TNF-α, IL-1β, IL-6 transcripts in freshly purified PBMCs from healthy controls (Ctrl) or the WS patient. Data were normalized to the expression of *ACTB* (encoding β-actin) and presented relative to results in healthy controls untreated cells (dotted line, 1-fold). In (**B**) and (**C**) data were analyzed by two-tailed Student’s *t*-test. ^*^P < 0.05; ^*^^*^P < 0.01; ^*^^*^^*^P < 0.001; ^*^^*^^*^^*^P < 0.0001. (**D**) Schematic heat map representation of Cytokine 25-Plex Human ProcartaPlex™ Panel 1B. The cytokine profile was analyzed in sera from healthy donors (Ctrl), type 1 diabetes subjects (T1D) and the WS proband.

A growing body of evidence suggests that ER stress may be a trigger of chronic inflammation and, conversely, inflammation is an important amplifier of ER stress (11–13). In addition, ER stress induction may also be involved in the induction of WFS1 (14,15). Although we could not detect a constitutive upregulation of UPR markers, we assessed whether the c.757A > T *WFS1* gene would impact the immune system. To this purpose, we cultured proband’s and control PBMCs for 48 h and measured a panel of cytokines in culture supernatants by multicytokine ELISA. Results clearly revealed that the PBMCs of the WS patient can secrete higher levels of several proinflammatory cytokines, particularly TNF-α, IL-1β and IL-6, as compared to healthy donors (Fig. 3A).

**Figure 4:**
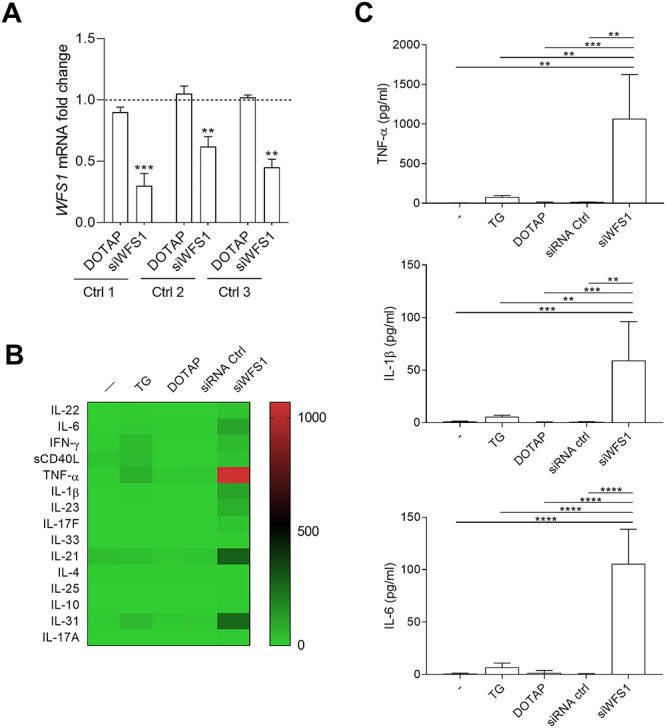
Production of proinflammatory cytokines after silencing of the *WFS1* gene in PBMCs from healthy donors. (**A**) The mRNA level of *WFS1* after transfection with *WFS1* siRNA was detected by real-time PCR in PBMCs of three healthy donors. Control cells were treated with vehicle alone (DOTAP). Data were normalized to the expression of *ACTB* (encoding β-actin) and presented relative to results in untreated cells (dotted line, 1-fold). One experiment is shown representative of three. Data were analyzed by one-way ANOVA followed by post hoc Bonferroni’s test. (**B**) Schematic heat map representation of Bio-Plex Pro Human Th17 cytokine panel. The cytokine profile was analyzed in supernatants from PBMCs of healthy donors untransfected or transfected with siWFS1 or a scramble siRNA. Vehicle alone (DOTAP) was used as a control. Individual expression levels of selected cytokines were reported in (**C**). Data were analyzed by one-way ANOVA followed by post hoc Bonferroni’s test. ^*^^*^P < 0.01; ^*^^*^^*^P < 0.001; ^*^^*^^*^^*^P < 0.0001.

In order to evaluate the statistical significance of these data, we analyzed the most relevant cytokines also by means of single-cytokine ELISA (Fig. 3B). Results showed that PBMCs bearing the c.757A > T *WFS1* gene significantly release 5–20-fold higher amounts of TNF-α, IL-1β and IL-6. Consistent with these data, significantly higher levels of TNF-α, IL-1β and IL-6 transcripts could be measured in the PBMCs of the WS proband as compared to controls (Fig. 3C). Higher levels of several cytokines, including TNF-α, IL-1β and IL-6, could also be detected in the serum of the WS patient as compared to healthy donors (Fig. 3D). An alteration in the concentrations of several proinflammatory cytokines was found also in sera of type 1 diabetes (T1D) patients. However, the cytokine profile between T1D and WS patients are not overlapping. Specifically, in the WS proband serum there were higher values of IL-1β, IL-6, TNF-α, GM-CSF and IL-27 than in T1D, whereas in the latter patients IFN-ɣ, IL-15 and IL-18 displayed higher concentrations than in the WS proband. Several cytokines were present at almost at the same levels in both the diseases, including IL-17A, IL-2 and IL-23. To investigate whether the misregulation of the cytokine profile could be directly ascribed to the loss of function of WFS1 and to exclude the possibility of other causative factors (i.e. the presence of DM in the WS patient), we transfected PBMCs of healthy donors with a small interfering RNA (siRNA) specific for *WFS1* (siWFS1) (Fig. 4A). Vehicle alone (DOTAP) and a scramble siRNA were used as a control. As shown in Figure 4B and C, *WFS1* silencing dramatically increased the production of several proinflammatory cytokines (Fig. 4B and C), to levels much higher (>100-fold) than those released by the PBMCs from the WS patient (Fig. 3A and B). Because WFS1 mutation/loss of function is responsible for neurodegeneration in WS, we also evaluated serum NfL (neurofilament light chain), MBP (myelin basic protein) and α-synuclein as validated biomarkers for neurodegeneration. We found that all these biomarkers values were comparable to those found in healthy donors (data not shown), probably because the proband does not have strong symptoms of neurodegeneration yet.

Overall, our data indicated that the loss of function of WFS1, either genetically predetermined as occurring in the WS patient or artificially induced in healthy PBMCs by gene silencing, will translate into a profile resembling a proinflammatory hypercytokinemia.

### Impact of the novel *WFS1* mutation on the WS proband adaptive immune system

**Figure 5:**
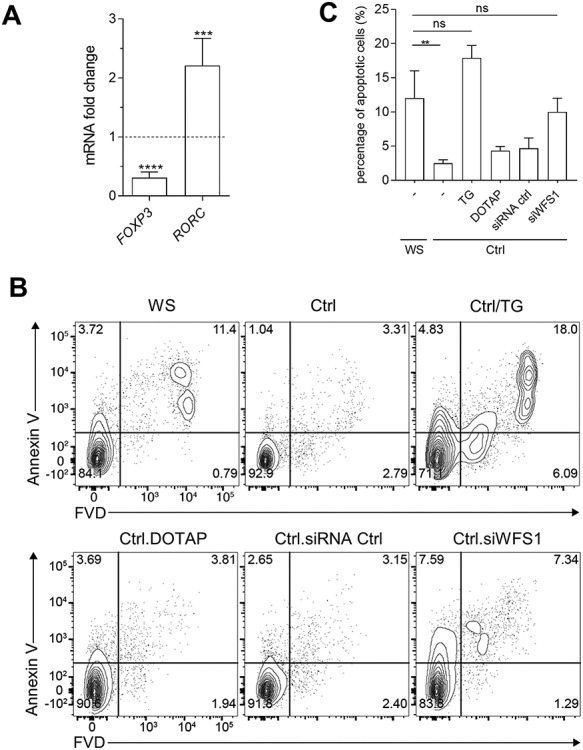
Cell dysregulation and apoptosis in proband’s PBMCs. (**A**) *FOXP3* and *RORGT* transcripts were analyzed by real-time PCR in WS PBMCs and healthy controls. Data were normalized to expression of *ACTB* (encoding β-actin) and presented relative to results in healthy controls PBMCs (dotted line, 1-fold). Data were analyzed by two-tailed Student’s *t*-test. (**B**) (**C**) *In vitro* evaluation of apoptosis. PBMCs of WS patient were compared to PBMCs of healthy controls (Ctrl) untreated, treated with TG at 0.5 μM or transfected with siWFS1, siRNA control, or the vehicle alone (DOTAP). Cells were cultured for 24 h and percentages of apoptotic cells were measured by flow cytometry. (**B**) The percentage of apoptotic cells was quantified as the percentage of positive cells in the gated area. (**C**) Quantification and statistical analysis of flow cytometry analysis plots, a representative one is in (**B**). Data were analyzed by one-way ANOVA followed by post hoc Bonferroni’s test. ns, not significant; ^*^^*^P < 0.01; ^*^^*^^*^P < 0.001; ^*^^*^^*^^*^P < 0.0001.

In several chronic diseases characterized by high basal levels of proinflammatory cytokines, an imbalance of specific T cell sub-population—namely, Th17 and T regulatory (Treg) cells—has been observed (16–18). These two cell types play opposite roles in immune responses (19). Th17 is a proinflammatory Th cell subset involved in physiological and pathological processes in various autoimmune and infectious inflammatory diseases (20), whereas Treg cells constitute a T cell subset that can maintain self-tolerance, suppress the activity of a variety of immune cells, and inhibit immune responses (21). Therefore, to gain a deeper insight into how c.757A > T *WFS1* gene could impact the immune system of the WS patient, we analyzed the percentages of circulating Treg and Th17 cells. Using PBMCs from both the WS patient and healthy donors, we analyzed transcripts coding for Foxp3 and RORγt, specific transcription factors of Treg and Th17 cells, respectively (22,23). Levels of Foxp3 and RORγt mRNA were markedly decreased (*P* < 0.0001) and increased (*P* < 0.001), respectively, in PBMCs from the WS patient as compared to healthy controls (Fig. 5A).

Studies previously reported that subjects with chronic inflammatory diseases (i.e. including autoimmune diseases) have decreased numbers and/or regulatory function of Treg cells than healthy control subjects (24–27), possibly due to apoptotic events favored by the presence of high levels of proinflammatory cytokines (28). Therefore, owing to the Th17/Treg ratio imbalance in peripheral blood of the WS proband and to the hyperproduction of proinflammatory cytokines, we hypothesized that the immune cells of the WS patient could have impaired vitality. To prove this hypothesis, after 24-hour incubation, we stained proband’s PBMCs with Annexin V-FVD to assess the percentage of cells in apoptosis. As a control, we used PBMCs from healthy donors either untreated, treated with TG, or transfected with siWFS1 to better mimic cells with WFS1 loss of function. Vehicle alone (DOTAP) and a scramble siRNA were used as controls for siRNA transfection. Our study revealed a spontaneous basal apoptosis in the proband’s PBMCs that was significantly higher than in healthy controls but comparable to controls after TG treatment or *WFS1* silencing (Fig. 5B and C). Overall, these data highlighted important alterations in adaptive immune cells of the WS patient as compared to healthy controls.

## Discussion

A large number of mutations in the *WFS1* gene have been identified so far, with the majority being located in the exon 8 coding for the nine transmembrane segments and the C-terminal tail of the WFS1 protein. Novel mutations are continuously found in this gene and many studies are conducted in order to understand their pathogenic role as well as their genotype/phenotype relationship (29–31).

In this study, we reported the occurrence of two novel *WFS1* mutations outside the exon 8, namely, c.316-1G > A (in intron 3, paternally inherited) and c.757A > T (in exon 7, maternally inherited), in a WS patient. Each one of these mutations was tolerated in heterozygous parents, indicating that the normal allele product compensates for the mutant one. On the contrary, the proband inherited both mutated alleles and this condition was associated with no wild type WFS1 protein production. By the use of the Human Splicing Finder and the Mutation Taster tools it was possible to predict that c.316-1G > A could cause intronic retention and/or exon skipping. Retention of the intron in the transcript could lead to a truncated WFS1 protein, because many introns contain in-frame premature termination codons (32), whereas skipping of exon 4 could produce a transcript coding for an aberrant protein. In any case, both of these putative splicing variants could undergo NMD. Particularly, by using a specific PCR strategy, we found that PBMCs of the WS patient lack both possible transcripts expected from the c.316-1G > A mutated allele. These findings suggest either that no intronic retention nor exon skipping occurs because the allele is not expressed, or that a mechanism of NMD causes transcript degradation. Instead, the PCR analysis encompassing exons 3–5 highlighted a 323 bp product consistent with either wild type and c.757A > T *WFS1*. However, no truncated protein possibly encoded by the c.757A > T mutated transcript was detectable, maybe because the short mutated WFS1 protein in proband’s PBMCs undergoes a proteasomal degradation process.

The connection between ER stress and WFS1 has been widely demonstrated (7) and pancreatic β-cells are particularly sensitive to ER stress than other types of cells and tissues (8,33). These findings could explain the reason why diabetes is usually the first symptom in WS, in which the occurrence of several mutations in the *WFS1* gene dampens its role in the control of the ER stress response. Interestingly, although we were not able to detect the WFS1 protein in our samples, there was no constitutive UPR activation in proband’s PBMCs. One possible explanation could be that in PBMCs, but not in other tissues such as pancreatic islets, there are proteins compensating for WFS1 loss of function, at least in terms of lack of control of the ER stress response. This is an interesting possibility that merits further investigations and that could be useful to overcome the effects of WFS1 deficiency in distinct tissues and organs involved in WS.

The existence of a bidirectional cross talk between the UPR signaling and inflammation has been described, and cell types that have metabolic or immune functions are extremely sensitive to alterations in metabolism and/or ER homeostasis (12). Therefore, ER stress can initiate the events leading to a disease and inflammation may be an important amplifier of ER stress (13). Although proband’s PBMCs with the mutated WFS1 did not show ER stress activation in basal conditions, they were found to constitutively secrete high levels of several proinflammatory cytokines, including TNF-α, IL-1β and IL-6. Moreover, by measuring cytokines in sera, a condition of hypercytokinemia could also be revealed in the WS patient. Such proinflammatory cytokine profile seems to be strictly correlated to the WS, rather than the comorbidity of DM that is present in the proband. In fact, the comparison of the serum cytokine profile between T1D patients and WS proband revealed that different cytokine networks are at work in orchestrating inflammation in T1D and WS. Further evidence that the cytokine profile found in the proband is strictly dependent on WFS1 competence was the finding that silencing of the *WFS1* gene in healthy donors PBMCs is responsible of an almost overlapping cytokine profile with the proband's PBMCs. This effect was specific for *WFS1* and not a general consequence of siRNA silencing of any gene. In fact, in the literature there are several examples demonstrating that specific gene silencing can increase or reduce the production of the proinflammatory cytokines, and it is strictly dependent on the specific siRNA sequence and the gene being silenced. In quantitative terms, however, WFS1 deficiency by silencing had a stronger impact on the production of proinflammatory cytokines than the mutated WFS1 in proband’s PBMCs. These findings may suggest again the existence of compensatory mechanisms in the proband’s PBMCs that, in this context, may avoid an acute, systemic shock.

The mechanisms responsible for the inflammatory cytokine levels increase in WS have not yet been investigated. However, considering the data from the current literature, it is possible to hypothesize that thioredoxin-interacting protein (TXNIP) could have a crucial role. Oslowski *et al.* reported that TXNIP is a key molecule connecting ER stress to inflammation (34). They found that TXNIP is a proapoptotic component of the ER stress response and is a critical link between ER stress and inflammasome activation. These data were confirmed in another work from Urano’s group, demonstrating that TXNIP is a key mediator of cytokine-induced β cell death in T1D (35). Noteworthy, TXNIP plays an important role in various types of immune cells as well and it seems to be required for the maintenance of immune cell function under normal physiological conditions (36,37). However, we observed that, although in the proband’s PBMCs there is not constitutive ER stress activation, these cells are more prone to ER stress activation. Whether this condition is sufficient to establish TXNIP upregulation in PBMCs remains to be investigated.

The WS patient displayed additional immune dysregulations, such as an imbalance of the Th17/Treg cell ratio, indicating the dominance of proinflammatory Th17 cells and thus inflammation, and an augmented apoptotic process. In several chronic inflammatory diseases, such as psoriasis (38), rheumatoid arthritis (18), uveitis (17) and hepatitis B-associated liver cirrhosis (16), a Th17/Treg dysregulation in the peripheral blood of the subjects has been observed and this alteration has been found to be involved in the pathogenesis of the disease. Moreover, Th17 cells are known to act through the secretion of multiple cytokines, including IL-17A and IL-17F, whose increase in the WS patient could indeed be observed in our cytokine profile analyses (39–41). Furthermore, several proinflammatory cytokines, such as IL-6, IL-1β and TNF-α, are inducers of Th17 cells in humans (42). In contrast, in many chronic inflammatory/autoimmune diseases, Treg cells have decreased regulatory function or are present at lower numbers than in healthy controls (24–27). Interestingly, previous studies revealed an increased spontaneous apoptosis of Treg cells in patients with inflammatory/autoimmune diseases, as T1D (28), autoimmune hepatitis (43), rheumatoid arthritis (44) and chronic inflammatory bowel disease.

In conclusion, our data suggested that WFS1 deficiency may cause effects other than alterations in UPR signaling pathways. More specifically, this condition can profoundly affect the immune system by promoting a systemic chronic inflammation and an imbalance in T lymphocytes involved in inflammation versus immune regulation. Therefore, it is reasonable to hypothesize that therapies blocking inflammatory cytokines, such as monoclonal antibodies specific for TNF-α and the IL-6 receptor, could be useful to reset systemic immunity and possibly other important manifestations of the disease in WS patients.

To our knowledge, this is the first study that describes an alteration in the cytokine profile in a patient with WS. However, it is possible to hypothesize that any mutation responsible of WFS1 loss of function can cause hypercytokinemia, because silencing of the *WFS1* gene in healthy donors PBMCs led to a cytokine profile almost overlapping with c.757A > T WFS1 PBMCs.

As a whole, these findings may be useful also for all WS patients. In fact, studies on WS have revealed the natural history, disease progression markers, and mechanisms of the disease, but a serum biomarker has not been identified yet. In this article, we show that inflammatory cytokines are potential serum biomarkers for WS. This is an important contribution to the efforts aimed at tracking the progression of the disease and to assess the efficacy of potential treatments. Moreover, these findings pave the way to the possibility that unexplained symptoms in WS, such as chronic fatigue and progressively declining levels of physical stamina, can be caused by high levels of systemic inflammatory cytokines. In fact, it is documented that cytokines present in both blood and cerebrospinal fluid are closely associated with the progression and severity of chronic fatigue syndrome, a disease characterized by disabling fatigue, depression, memory loss and somatic discomfort (45).

Although WS is a rare disease, the study of this syndrome is becoming more and more important. In fact, both *WFS1* and *WFS2* (namely, the gene responsible for WS2, the rarer form of WS) seem to be directly and/or indirectly involved in the pathogenesis of several neurodegenerative diseases. A recent excellent review reported that Alzheimer’s disease shares common mechanisms with WS (46). Particularly, deficiency of *WFS1* increases neurodegeneration in both Drosophila overexpressing human tau and in an Alzheimer’s disease-like mouse model, respectively (47). Furthermore, *WFS1* polymorphisms and mutations have been associated with Parkinson’s disease as well as with a variety of psychiatric illnesses (48,49). Overall, this information highlights the importance of characterizing WS and underlines that new possible therapies as well as current ongoing clinical trials targeting WS may also provide very useful insights for developing novel therapeutic strategies against other diseases without a cure, such as Alzheimer’s disease.

## Materials and methods

### Patient and family history

Our case-study concerns a 11-year-old female, the older of two children, with familial history of DM (grandfather with type 2 DM, second cousin with type 1 DM). No family member was suffering from psychiatric symptoms, visual disorders or hearing loss. The parents are healthy and non-consanguineous. All family members showed no signs of WS. She was diagnosed as affected by insulin-dependent DM without autoantibody when she was 5 years old, with onset symptoms characterized by polyuria, polydipsia, polyphagia, astenia and glycosuria. At the age of 9 years, she developed initial signs of OA recognized during follow-up visits and WFS was suspected. Consequently, whole exome sequencing was performed.

### Cell purification

Peripheral venous blood (3–5 ml) was obtained from the proband, T1D subjects (*n* = 5) and age-matched children and adults (*n* = 5) (i.e. those without T1D or any autoimmune or allergic disease) attending the Day Service of San Raffaele Hospital (Milan, Italy). Informed consent for the studies was obtained. PBMCs were isolated on a Ficoll–Hypaque gradient from the proband and healthy subjects. PBMCs (1 × 10^6^ cells/ml) were cultured for 48 h *in vitro* in complete medium (RPMI with 10% FCS, 10 mM HEPES and 50 μM 2-ME), in the presence or absence of 0.5 μM TG (Sigma). Sera were obtained by blood centrifugation at 450 × *g* for 5 min and frozen at −80°C in criovials until analyses.

### Nucleic acids isolation, PCR and real-time PCR

A panel of genes associated with monogenic diabetes was sequenced at San Raffaele Hospital by using the TruSight One sequencing panel (Illumina, San Diego, USA). Genomic DNA (gDNA) extraction from PBMCs was performed using the ‘salting out’ method. Briefly, frozen cell pellets were thawed and lysis buffer (100 mM Tris, 200 mM NaCl, 5 mM EDTA, 0.2% SDS) and proteinase K were added. After an incubation of 2 h at 45°C, ethanol was added and samples were centrifuged (1300 rpm for 15 min at 4°C). Genomic DNA pellets were washed with 70% ethanol and resuspended in water. Total RNA was isolated using the Trizol Reagent (Thermo Fisher Scientific, USA) according to manufacturer’s instructions. RNA was quantified and 1 μg of RNA was used for complementary DNA (cDNA) synthesis through QuantiTec Reverse Transcription kit (Quiagen, Germany) according to manufacturer’s instructions.

To evaluate the presence of the predicted mutations, an end-point PCR on cDNA samples and on gDNA samples as a control was performed using the following primers: forward 5′-TTGAAGAAGTCCTGGAGAGG-3′; reverse 5′-CACCTGCTTCTTCTTCTTGG-3′. To assess cDNA sample normalization, an end-point PCR for the housekeeping gene *ACTB* (coding for human β-actin) was performed. Primers sequence is indicated in Table 1. PCR products were run on 1% agarose gels with EtBr, in TAE (tris acetate EDTA) buffer. Quantitative PCR for specific genes was performed using SYBR green dye (Bio-Rad CA, USA). Primers used for real-time PCR analysis are listed in Table 1. Data were presented as the ratio of gene expression to *ACTB* (coding for human β-actin) expression determined by the relative quantification method (ΔΔCT).

**Table 1:** Primers used for quantitative real-time (RT)–PCR

Genes	Forward	Reverse
*CHOP*	5′-CAGAACCAGCAGAGGTCACA-3’	5′-AGCTGTGCCACTTTCCTTTC-3’
*sXBP-1*	5′-CTGAGTCCGCAGCAGGTG-3′	5′-TGCCCAACAGGATATCAGACT-3’
*ATF4*	5′-GACCGAAATGAGCTTCCTGA-3′	5′-ACCCATGAGGTTTGAAGTGC-3’
*GRP78*	5′-CACAGTGGTGCCTACCAAGA-3′	5′-TGATTGTCTTTTGTCAGGGGT-3’
*TNFA*	5′-CTTTGGAGTGATCGGCCCC-3′	5′-GTTATCTCTCAGCTCCACGCC-3’
*IL1B*	5′-CAGAAGTACCTGAGCTCGCC-3′	5′-AGATTCGTAGCTGGATGCCG-3’
*IL6*	5′-CCACTCACCTCTTCAGAACGAATT-3’	5′-AGTGCCTCTTTGCTGCTTTCA-3’
*FOXP3*	5′-TGTGGGGTAGCCATGGAAAC-3′	5′-TCATTGAGTGTCCGCTGCTT −3’
*RORC*	5′-AGGCCATTCAGTACGTGGTG-3′	5′-TGCCACCGTATTTGCCTTCA-3’
*ACTB*	5′-CTCGTCGTCGACAACGGCT-3′	5′-TCAGGGTGAGGATGCCTCTC-3’

### Western blot analysis

Cells were washed twice in cold PBS and centrifuged. The protein pellet was dissolved in 1% SDS by incubating the sample at 50°C for 1 h and followed by centrifugation at 10 000 × *g* for 10 min at 4°C to sediment any insoluble material. Equal amounts of protein from each sample were solubilized in Laemmli sample buffer and heated for 5 min at 95°C. Proteins were separated by SDS-PAGE on 10% polyacrylamide gels and then transferred to nitrocellulose. Membranes were blocked with 5% (wt/vol) non-fat dried milk in tris-buffered saline containing 0.1% (vol/vol) Tween 20 at room temperature for 1 h. After being blocked, membranes were incubated overnight with primary antibodies, namely, rabbit anti-WFS1 (Cell Signaling Technologies) and mouse anti-β-tubulin (Sigma), then washed and incubated with appropriate horseradish-peroxidase (HRP)-conjugated secondary antibody (Pierce) for 1 h at room temperature. Immunoreactive bands were detected by enhanced chemiluminescence (Bio-Rad, CA, USA).

### *WFS1* silencing and transfection

The *WFS1* gene was silenced by means of short interfering RNAs (siRNA) predesigned and inventoried Silencer Select siRNAs (Life Technologies) specific for human *WFS1* gene, siRNA IDs s14856. Silencer Negative Control siRNA (siCONTROL Nontargeting Pool, D-001810-10-05, Dharmacon) was used to determine transfection efficiency and controlling the effects of siRNA transfection. For transfection, siRNAs (5 μg) in 30 μL of transfection buffer (20 mM HEPES, 150 mM NaCl, pH 7.4) were pipetted into a sterile Eppendorf tube. In a separate polystyrene tube, 6.7 μg of 1,2 dioleoyl-3-trimethylammonium-propane (DOTAP) was mixed with 30 μl of transfection buffer and then both solutions were mixed gently by pipetting several times. After incubation at room temperature for 20 min, the mixture was added to 1 ml of complete medium containing 1 × 10^6^ cells and incubated for 48 h at 37°C. Cells were recovered, washed, and used for experiments.

### Cytokine determinations

The cytokine profile was analyzed in cell culture supernatants by using a 15-plex immunoassay (Bio-Plex Pro Human Th17 cytokine panel; Bio-Rad, CA, USA) or in sera by using a Cytokine 25-Plex Human ProcartaPlex™ Panel 1B (Invitrogen) and a MAGPIX system (Luminex Corporation). ELISA kits for TNF-α, IL-1β (both Life Technologies), and IL-6 (eBioscience Inc.) were used to measure cytokines in 100 μl of cell culture supernatants according to the manufacturers’ protocol. Cytokine levels are represented as the mean ± standard deviation (SD) of the concentrations (pg/ml).

### Analysis of apoptosis

PBMCs derived from the proband and healthy controls as untreated, treated with TG at 0.5 μM or transfected with siWFS1, control siRNA, or vehicle alone (DOTAP), were incubated at 37°C and harvested after 24 h. Then, cells were collected by centrifugation, washed with PBS, stained with Fixable Viability Dye eFluor® 780 (eBioscience, CA) for 30 min and then washed with PBS and Flow Cytometry Staining Buffer. Five microliters of PerCP-eFluor 710-conjugated Annexin-V (eBioscience, CA) were added and incubated with cells at room temperature for 15 min. Percentages of PerCP-eFluor 710-positive apoptotic cells were determined by flow cytometry. Samples were run on LSRFortessa (BD Biosciences) flow cytometer and analyzed using the FlowJo analysis software (Tree Star). Each experimental point was performed in triplicates.

### Statistics

Data are representative of at least three independent experiments. All results are shown as mean ± SD. A two-tailed Student’s *t*-test was used to compare two treatments, whereas one-way ANOVA followed by post hoc Bonferroni’s test was used when three or more samples were under comparison. All analyses were performed using Graph Pad Prism software 8.0.

## Study approval

WS proband’s, T1D’s, healthy control children’s parents and healthy control adults provided informed written consent for the collection of samples and subsequent analysis.

## Authors’ contributions

MTP designed research; E Panfili, EO, E Proietti, AI and MTP performed experiments; GM, CO, MLB, MG, FF, P Prontera, E Proietti, CV and MTP analyzed data; GF and ET contributed in the clinical diagnosis of the proband; E Panfili, MLB, SE, UG and P Puccetti contributed writing parts of the manuscript; MTP wrote the paper. All Authors approved the final version of the manuscript.

## Supplementary Material

Supplementary_figures_ddab040
