# Supplementary material for: Novel mutations in the WFS1 gene are associated with Wolfram syndrome and systemic inflammation
Source: Hum Mol Genet. 2021 Mar 9;30(3-4):265–76. doi: 10.1093/hmg/ddab040 (PMC8091036; doi:10.1093/hmg/ddab040)
Supplement: Supplementary_figures_ddab040 [file supplementary_figures_ddab040.pdf]

A novel mutation in the *WFS1* gene is associated with Wolfram syndrome and systemic inflammation

Supplementary Figures

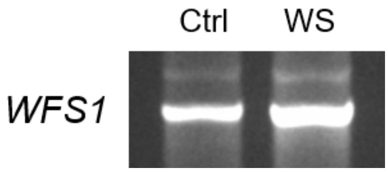

Figure Supplementary 1. PCR analysis to validate the primers. Genomic DNA amplification of both healthy donors (ctrl) and WS patient was performed using primers annealing on exon 3 and exon 5.

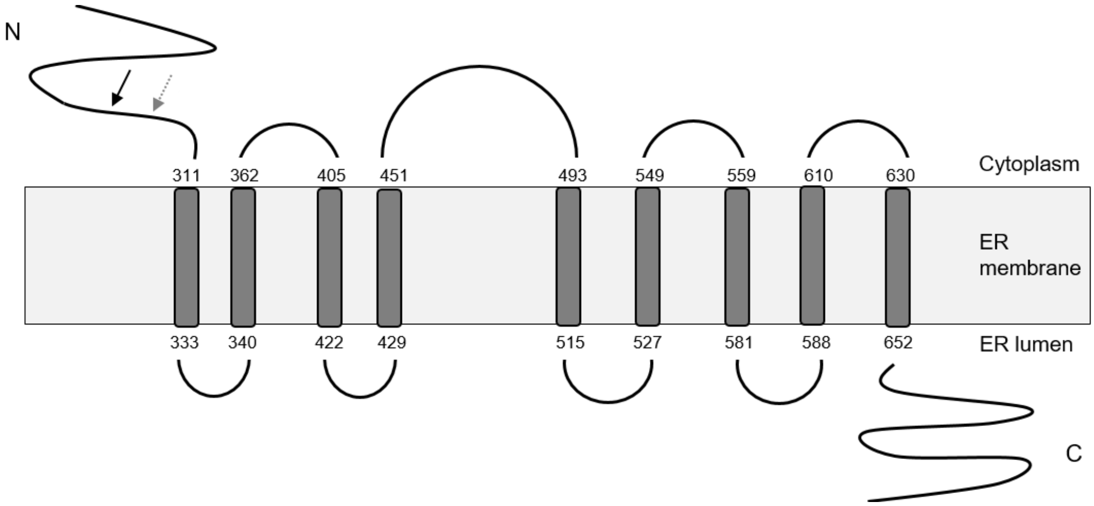

Figure Supplementary 2. Localization of the two novel mutations in WFS1 protein. Both the mutation in intron 3 (black arrow) and the one in the exon 7 (grey dotted arrow) are responsible for stop signal at codon localized in the N-terminal part of the protein.
